# A Machine Learning Approach to Identify Clinical Trials Involving Nanodrugs and Nanodevices from ClinicalTrials.gov

**DOI:** 10.1371/journal.pone.0110331

**Published:** 2014-10-27

**Authors:** Diana de la Iglesia, Miguel García-Remesal, Alberto Anguita, Miguel Muñoz-Mármol, Casimir Kulikowski, Víctor Maojo

**Affiliations:** 1 Biomedical Informatics Group, Dept. Inteligencia Artificial, Escuela Técnica Superior de Ingenieros Informáticos, Universidad Politécnica de Madrid, Boadilla del Monte, Madrid, Spain; 2 Department of Computer Science, Rutgers – The State University of New Jersey, Piscataway, New Jersey, United States of America; University of Cyprus, Cyprus

## Abstract

**Background:**

Clinical Trials (CTs) are essential for bridging the gap between experimental research on new drugs and their clinical application. Just like CTs for traditional drugs and biologics have helped accelerate the translation of biomedical findings into medical practice, CTs for nanodrugs and nanodevices could advance novel nanomaterials as agents for diagnosis and therapy. Although there is publicly available information about nanomedicine-related CTs, the online archiving of this information is carried out without adhering to criteria that discriminate between studies involving nanomaterials or nanotechnology-based processes (*nano*), and CTs that do not involve nanotechnology (*non-nano*). Finding out whether nanodrugs and nanodevices were involved in a study from CT summaries alone is a challenging task. At the time of writing, CTs archived in the well-known online registry ClinicalTrials.gov are not easily told apart as to whether they are *nano* or *non-nano* CTs—even when performed by domain experts, due to the lack of both a common definition for nanotechnology and of standards for reporting nanomedical experiments and results.

**Methods:**

We propose a supervised learning approach for classifying CT summaries from ClinicalTrials.gov according to whether they fall into the *nano* or the *non-nano* categories. Our method involves several stages: i) extraction and manual annotation of CTs as *nano* vs. *non-nano*, ii) pre-processing and automatic classification, and iii) performance evaluation using several state-of-the-art classifiers under different transformations of the original dataset.

**Results and Conclusions:**

The performance of the best automated classifier closely matches that of experts (AUC over 0.95), suggesting that it is feasible to automatically detect the presence of nanotechnology products in CT summaries with a high degree of accuracy. This can significantly speed up the process of finding whether reports on ClinicalTrials.gov might be relevant to a particular nanoparticle or nanodevice, which is essential to discover any precedents for nanotoxicity events or advantages for targeted drug therapy.

## Introduction

Because of their nanoscale biophysical and biochemical interaction characteristics, products containing nanomaterials or involving the application of nanotechnology behave very differently than traditional bulk materials used in therapeutics [1]. For instance, on the positive side, nanotechnology products can have increased bioavailability and potency, decreased toxicity and the enabling of highly targeted drug delivery, among many other advantages [2], making them potentially valuable for medical applications. However, experimental research in nanomedicine is still in its infancy, and many nanoparticles and nanotechnologies have the potential of also producing complex and poorly understood toxic side-effects [3]. As result, discovering and reporting of *in vivo* effects of nanoparticles in experimental studies and CTs to supplement theoretical predictions and results of *in vitro* studies [3], [4] is fundamental to advance the discovery and use of nanomaterials as beneficial agents for medical diagnosis and therapy. Clinical Trials (CTs) of nanoparticles and nanodevices may lead to novel treatments and diagnostic tools, as well as complement or supplement existing products that are currently being nanomanufactured to address clinical needs (e.g. effectiveness) while avoiding the undesirable side-effects (e.g. toxicity) [5], [6].

Over the past years, there has been a proliferation of pre-clinical and clinical studies on nanopharmaceuticals [7]. Unfortunately, online archiving of nanomedical clinical data has been usually done without following any specific criteria, thus not making a distinction between those studies at the nanoscale focused on testing nanodrugs and nanodevices (*nano* CTs) and CTs on regular drugs and biologics that do not involve nanotechnology (*non-nano* CTs). Thus, it is becoming difficult to develop a broad and comprehensive perspective on the current status and impact of nanotechnology products in the clinical context.

A number of articles have analyzed published trial results in terms of quality and completeness [8], [9], as well as the impact of the public release of CTs [10]–[12], but these and other studies have not yet made, to our knowledge, such a clear distinction between *nano* and *non-nano* categories either. Recent publications in the scientific literature have reviewed specific medical applications of nanotechnology-based pharmaceuticals, but these publications only reported a few specific nano-level products currently reported positively in CTs [7], [13]–[14] and certain nanodrug patents [15]–[17]. We believe that the automatic annotation of CT reports to tell whether they are *nano* or not, can be of great help to clinical researchers in nanomedicine, and we will show in this paper that such a classification can come from comprehensively searching for, and detecting the presence of nanodrug and nanodevice involvement in a study from the CT summaries published in public registries, such as ClinicalTrials.gov.

The analysis of nanoinformation—a new field, to which we have already contributed [18]–[21]—suggests that data resulting from research in nanomedicine are highly heterogeneous, and distributed among many data sources, without usually following any specific data standard or using a controlled vocabulary [22]. Since the volume of nanomedicine experimental and clinical data is increasing rapidly, manual searches, and subsequent analyses and annotation of studies on nanodrugs and nanodevices, does not scale, and becomes increasingly infeasible [23]. In this paper, we present a novel approach for the analysis of summaries of CTs, designed to detect the mention of nanotechnology products as test targets, as opposed to the testing of conventional drugs in a trial, so as to demonstrate the viability of applying automatic methods to carrying out the detection, and suggesting, by our cross-validation results, that the techniques can be extended to predictive detection and annotation of the CTs.

### Clinical Trials for Nanomedicine

A major issue when analyzing a nanomedical text is how to define the term “*nano*” [24]. Many attempts to characterize nanotechnology can be found in the literature [25] but a standard or consensus definition—proposed or accepted by all the regulatory authorities in the field—has yet to be established. For instance, the International Organization for Standardization (ISO) and the US National Nanotechnology Initiative (NNI) defines nanotechnology as “the understanding and control of matter at dimensions between approximately 1 and 100 nanometers, where unique phenomena enable novel applications” [26], [27]. In addition, there exist nanomedical studies which include materials at higher scales, e.g. considering structures with at least one dimension that reaches up to 300 nanometers [7], or 500 nanometers [28], or, at the lower end of the scale, those that are even smaller than 1 nanometer [29], [30].

Criteria that can distinguish between *nano* and *non-nano* drugs and devices are still under discussion, suggesting the urgent need for ways to define what are nanomaterials and nanotechnology products from their existing descriptions in the literature and in CT reports. For instance, in Europe, a broad range of characteristics—such as the size distribution or the volume-specific surface area—is used to define a nanomaterial [31], [32], even though size is still the key element for distinguishing products at the nanoscale. European recommendations consider not only single or primary particles but also agglomerates—“clusters of molecules or particles resulting from a process of contact and adhesion whereby dispersed molecules or particles are held together by weak physical interactions, which can be dispersed again” [33]—, aggregates—“clusters of chemically-bound nanoparticles held together by strong chemical or sinter forces through a non-reversible process” [33]—, and structured particles, but do not apply any universal threshold to their size. The most recent definition [34] considers that a nanomaterial should fulfill at least one of the following conditions: i) consists of particles, with at least one external dimension in the size range 1–100 nm for more than 1% of the number size distribution; ii) has internal or surface structures in at least one dimension in the size range 1–100 nm; and, iii) has a specific surface area by volume greater than 60 m^2^/cm^3^. In the USA, according to the Food and Drug Administration (FDA), nanotechnology products must have at least one dimension in the nanoscale (1–100 nanometers) or, in the case of products with a size range of up to one micrometer, they must exhibit properties and phenomena that are attributable to their dimension(s) and distinct from those of macroscale materials [35]. Consequently, researchers use different measures and properties to distinguish between *nano* and *non-nano* products, ranging from size, size distribution and surface area, to other material properties, such as crystalline phase, photocatalytic activity, zeta potential, or water solubility, among many others.

### Online Clinical Trial Registries

Full disclosure of CT outcomes is essential to avoid duplication of efforts in clinical research, as well as bias in the publication of results—for instance, selective reporting based on the commercial interests or failure to report important adverse events [36]. In most cases, pharmaceutical and commercial data are confidential and, therefore, additional and more effective sharing policies on clinical results are still needed.

Registration and public access to CT results have been frequently discussed [37]–[41]. In 2004, the International Committee of Medical Journal Editors (ICMJE) published a statement establishing the disclosure of clinical trial results in public registries as a requirement for their publication in scientific journals [42]. Staggered public release of CT results started in 2007 after several legal rulings [43]–[45] were made regulating public access to CT summaries. Nowadays we can find numerous databases offering free online access to data reported about CTs, such as, for instance, the ClinicalTrials.gov database (http://clinicaltrials.gov/) in the USA [46], the EudraCT database accessible through the European Union Clinical Trials Register (https://www.clinicaltrialsregister.eu/) [47] and the Japan Medical Association Clinical Trials Register (JMACTR) (https://dbcentre3.jmacct.med.or.jp/jmactr/Default_Eng.aspx/). There is still no single comprehensive international registry of CTs but the International Clinical Trials Registry Platform (ICTRP) (http://apps.who.int/trialsearch/Default.aspx/), developed by the World Health Organization (WHO), includes these databases, while offering a uniform access mechanism to clinical trial data stored on them [48]. There are also several public repositories focused on specific diseases. For instance, the National Cancer Institute provides an online searchable database (http://www.cancer.gov/clinicaltrials/search/) with about 37,000 cancer-related CTs. Additionally, some databases owned by the pharmaceutical industry can also be accessed through the Internet, such as the GlaxoSmithKline Clinical Study Register (http://www.gsk-clinicalstudyregister.com/; https://clinicalstudydata.gsk.com/) [49]. Even though all of these are valuable resources for clinical researchers in the biomedical and nanomedical areas, none of them makes an explicit and clear category distinction between CTs supporting *nano* and *non-nano* products.

**Figure 1:**
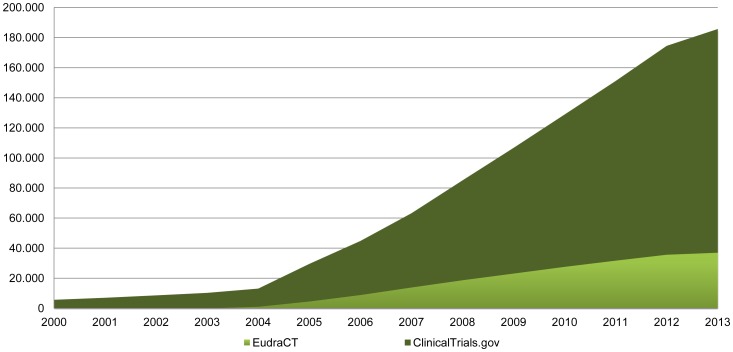
Total number of registered clinical studies in EudraCT and ClinicalTrials.gov over the last years.

The work presented in this paper analyzes CT summaries extracted from the ClinicalTrials.gov database. Since its release in 2010, the ClinicalTrials.gov database has grown considerably (Figure 1) and several tools have been developed to extend and improve its functionalities [50], [51]. At the time of writing, the database provides access to about 150,000 registries of CTs conducted in 185 different countries, including clinical studies on nanoparticle formulations and medical products containing nanomaterials and nanodevices. We selected this database among many others due to its volume of data, its scope and quality, as well as its programmatic interface, which allows users to download study record data in a machine-readable format (eXtensible Markup Language, XML) that can be easily analyzed.

### Overall aim of this work

In this paper, we present a novel approach to the classification of summaries of CTs between those that were targeted at testing nanotechnology products *vs*. those targeted at testing conventional drugs, and demonstrate the feasibility of applying automatic methods to produce such a classification for the purpose of annotation and indexing. The obtained results are promising, suggesting that the CT summaries archived in ClinicalTrials.gov do contain the required information for a machine learning method to automatically identify whether the summary refers to a CT involving nanoparticles and/or nanodevices or not. The possibility of automatically distinguishing CTs that support *nano* vs. those that support *non-nano* products is timely and necessary due to the growing information challenges posed by nanomedical research which make manual comprehensive detection of *nano* trials from CT summaries by experts increasingly difficult and costly, as well as tedious and error-prone.

## Materials and Methods

To address the classification problem described above, we identified, selected and extracted 500 nanodrug-focused CTs from ClinicalTrials.gov. In addition, we created a control group composed of 500 CTs not involving any nanodrugs or nanodevices. Both sets define our training and test set, and form the baseline reference set composed of documents correctly labeled as *nano* and *non-nano* (Table S1). We applied state-of-the-art machine learning methods —reported in the scientific literature to be suitable for the document classification task, as we will see next— to generate multiple classification models that were assessed using cross-validation (both 10-fold and leave-one-out cross validation).

This section describes the proposed approach in detail. First, we define the scope of our study as well as the procedure we followed to identify, select and gather the CT summaries to build the training and test set. Then, we describe the different methods we used to conduct the experiments, including the data pre-processing techniques and the machine learning algorithms that we applied for document classification. The section concludes with a description of the validation processes we used to compare the performance of the generated models for classifying CTs.

### Scope of the analysis

We manually selected a representative set of CT summaries from the ClinicalTrials.gov repository, based on the criteria and procedures described next. First, we conducted a simple search browsing studies by keyword: i) those CTs containing the term “nano” (for which 55 results were found) and, ii) those CTs containing the term “nanoparticle” (which yielded 133 results). It should be noted that the ClinicalTrials.gov database does not allow searching by prefix, which prevented us from searching all the terms that start with the prefix “nano”. In order to make this first search as general as possible and avoid the use of terms related to a specific drug, we selected the words “nano” and “nanoparticle” as search terms. Such as small total of 188 CT registries manually validated and labeled as *nano*, considering the numerous nanomedical products that are currently present in CTs, suggests that detecting the use of nanodrugs and nanodevices as being involved in a CT by using simple keyword searches on their summary descriptions may not yield all documented examples of this type of CT.

Therefore, to detect the presence of concepts and semantic entities that might be related to the nanomedicine field within the registries of the ClinicalTrials.gov database, we enriched our set of search terms by including specific concepts from an external source developed by the National Cancer Institute, the NCI Metathesaurus [52]. The NCI Metathesaurus combines a large number of clinically and biologically relevant controlled vocabularies and terminologies (http://ncim.nci.nih.gov/ncimbrowser/pages/source_help_info.jsf), some directly related to nanotechnology and, specifically, to nanomedicine, such as the NanoParticle Ontology (NPO) [53]. The goal of this step was to select the most descriptive terms that appear in nanomedical studies from this broad set of sources. For this purpose, we searched the terminology database for those entries containing the term “nano” in any field. This search returned more than 800 related biomedical concepts that were manually filtered afterwards, selecting only those concepts that belonged to one of the five semantic types considered potentially relevant to the nanomedical domain: Organic Chemical; Pharmacologic Substance; Inorganic Chemical; Biomedical or Dental Material; and, Indicator, Reagent, or Diagnostic Aid. Other semantic types were disregarded due to their lack of relevance for our study—e.g. Amphibian, Natural Phenomenon or Process, Plant, Eukaryote, etc. A total of 168 concepts were retrieved and validated, from which we obtained 433 terms by including all the synonyms pointed to by the Metathesaurus. We followed a dictionary-based approach by using the selected concepts provided by the Metathesaurus as input—i.e. as keywords—for conducting a new set of searches within the ClinicalTrials.gov database. Only 38 concepts (190 terms) extracted from the Metathesaurus returned results and were matched to clinical summaries. With this process, we retrieved a total of 344 CT registries that we validated and annotated with the label *nano* after a preliminary manual verification by us.

Using this combination of searches, we increased the set of potentially *nano* CT summaries to 414. This set was then analyzed in-depth: for each nano candidate, we conducted a comprehensive study of the intervention involved, checking out external data about the active component of the drug, its manufacturing process and the routes of administration. Targeted searches in diverse Web sources, such as PubMed (http://www.ncbi.nlm.nih.gov/pubmed/), the Web of Knowledge (http://wokinfo.com/), the Cochrane Library (http://www.thecochranelibrary.com/), Google, and a number of CT online registries, were carried out to retrieve nanodrug descriptions, which led to a myriad of sources. The scientific literature, for instance, provided us with a wealth of descriptions, usually reported as free-text entries. The authors also consulted a number of additional sources, including drug databases—e.g. Drugs@FDA (http://www.accessdata.fda.gov/scripts/cder/drugsatfda/), DailyMed (http://dailymed.nlm.nih.gov/dailymed/about.cfm/), TOXNET (http://toxnet.nlm.nih.gov/index.html/), ChemIDplus (http://chem.sis.nlm.nih.gov/chemidplus/)—, nanomaterials databases—e.g. Nanomaterial Registry (https://www.nanomaterialregistry.org/) [54]), pharmaceutical registries—e.g. Clinical Trials Portal of the International Federation of Pharmaceutical Manufacturers & Associations (http://clinicaltrials.ifpma.org/clinicaltrials/no_cache/en/clinical-trial-advanced-search/index.htm/)—, manufacturer websites—e.g. COMAR International database for certified reference materials (http://www.comar.bam.de/en/), European Reference Materials online catalogue (http://www.erm-crm.org/Pages/ermcrmCatalogue.aspx/), BAM Federal Institute for Materials Research and Testing list of nanoscaled reference materials (http://www.nano-refmat.bam.de/en/)—, patent records—e.g. PATENTSCOPE (http://www.wipo.int/patentscope/en/), Patent Full text Databases of the US Patent and Trademark Office (http://patft.uspto.gov/)—, and CT directories developed by advocacy organizations—e.g. American Association for Cancer Research SU2C Clinical Trials Finder (http://www.emergingmed.com/networks/AACR-SU2C/), International Myeloma Foundation trials searcher (http://myeloma.org/ResearchMatrix.action?tabId=26&menuId=0&queryPageId=14). Further, related CTs extracted from ClinicalTrials.gov, EUCTR, ICTRP and other sources, were also checked for comparison with a wide set of pre-clinical drugs. Information from all of these sources were used to cross-check and verify the nature of the drugs, formulations and medical devices identified as nanodrugs or nanodevices within the set of CTs earlier retrieved from ClinicalTrials.gov. From the analyzed information, we found a set of widely-used terms related to nanomedicine that can be considered as text patterns in clinical trials—e.g. liposomal, micelle, nanomaterial, nanosuspension, nanocolloid, crystal, nanotubes, gel, PEG, etc. These terms were also used to complete our search criteria and perform new searches in the ClinicalTrials.gov database that allowed us to retrieve additional clinical trial registries to complete the *nano* body of CT summaries. This body of documents was further double-checked by experts in the field of nanotechnology— Prof. Alejandro Pazos and Prof. Julián Dorado (University of A Coruña, Spain), as external experts, and Prof. Víctor Maojo and Prof. Casimir Kulikowski (co-authors)—, eliminating CTs that did not belong to nanomedicine and disregarding incomplete and inaccurate entries in the registries. Finally, the CTs involving *non-nano* products were randomly selected—i.e. without performing any specific query in ClinicalTrials.gov—and subsequently reviewed yet again to remove those CTs involving any relation to nanodrugs or nanodevices—with the few thus removed then becoming candidates that were considered to augment the set of CT *nano* entries. As result, we ended up with balanced training and testing sets, including 500 *nano* CTs and 500 *non-nano* CTs. For computational purposes, we downloaded the selected registries from the ClinicalTrials.gov website and stored all the study record data locally. Each record, in XML format, was processed with the Python library *lxml* (http://lxml.de/) to extract its textual content.

Through the procedure described above, we obtained the set of 1000 classified summaries, divided into the two different classes of *nano* and *non-nano*, depending on whether they were relevant or not to the targeting of nanoparticles or nanomaterials, or nanotechnologies, and hence falling into the nanomedical domain. This set of summaries (Table S1) provided the basis for the development and training of several types of classifiers, as described in the following subsections.

### Data Pre-processing

Data pre-processing is a critical step for improving classifier performance, which strongly depends on the data used to train the models [55]. To apply machine learning techniques and facilitate knowledge discovery during training, we first pre-processed all the documents (CT summary records) to eliminate irrelevant information from them. We performed several data standardization and filtering steps before running the data analysis, as detailed below.

For each entry (CT record), the textual content was tokenized and pre-processed using the Natural Language Toolkit (nltk) package for Python (http://nltk.googlecode.com/svn/trunk/doc/api/nltk-module.html) [56]. First, we split plain text into tokens by resorting to regular expressions: blank characters and punctuation marks provided the initial division into tokens. Tokens are defined as sequences of alphanumerical characters that may also include the underscore character (‘_’). Next, we replaced all the digits in the text by an arbitrary character (‘#’) and converted all text to lowercase. Stop words and short textual features were removed from the text, disregarding those tokens with less than three characters, as recommended in [57]. Then, we performed word stemming using Porter's algorithm for English [58], grouping words belonging to the same family by reducing them to their common stem.

Next we converted the plain text into textual features—unigrams and bigrams. A unigram can be defined as a single token extracted from the text while bigrams can be regarded as pairs of consecutive tokens found in the text. Each CT was represented as a vector of features implementing a “bag-of-words” approach [59], each component of the vector being either a unigram or a bigram. Table 1 shows a summary of the number of features—unigrams and bigrams—identified in the documents to illustrate their average size. For all documents in the collection, a total number of 11,164 unigrams and 38,124 bigrams were found. Each unigram occurred, on average, in 24 documents, while each bigram appeared in 6.3 documents. Related graphical summaries of results are provided in the Supporting Information, in Figures S1 and S2. These figures show that the distribution of N-grams per document —both unigrams and bigrams— is not normal, with a median of the distribution being 522,875 (unigrams per document) in the case of unigrams, and 297,667 (bigrams per document) in the case of bigrams.

**Table 1:** Statistics related to the number of features found in the body of CT summary documents.

	Unigrams		Bigrams	
	Number of unigrams	Number of unique(*) unigrams	Number of bigrams	Number of unique(*) bigrams
**Minimum number of N-grams per document**	111	66	45	31
**Maximum number of N-grams per document**	15092	1277	13124	1449
**Average number of N-grams per document**	732.462	268.282	450.179	240.772
**Standard deviation**	1000.856	138.014	788.709	167.367

The “Minimum number of N-grams per document” and the “Maximum number of N-grams per document” refer to the number of N-grams (both allowing and not allowing double- count) found in the documents containing the smallest and greatest number of N-grams in the collection, respectively. For instance, regarding unigrams, the document containing the smallest number of unigrams allowing double-count contains 111 unigrams, while the document containing the smallest number of unigrams not allowing double-count contains 66 unigrams.

(*) no double-count allowed

As stated above, we adopted a vector-based representation for the documents. Therefore, each document can be regarded as a vector with each of its components corresponding to a unique feature —unigram or bigram— in the collection. We built the features vector for our collection of 1000 documents using different representations:

1. *Binary representation*: each component of the vector denotes whether a unigram or bigram is present (1) or not (0) in the CT represented by the vector.
2. *Frequency-based representation*: in this representation we record the frequency of appearance of each feature (i.e. unigram or bigram) in the document.
3. *Inverse Document Frequency-based (IDF) representation*, which eliminates those terms that appear in too many documents, being therefore unlikely to discriminate well between the classes.
4. Term Frequency*Inverse Document Frequency-based (TFIDF) representation: Using this transformation it is possible to combine the local discrimination power of a term—i.e. in the context of a single document— with the global discrimination power of the term—in the context of the whole collection [60].

Finally, we also chose to normalize the resulting feature vectors. We applied a normalization step to all the representations above: each document vector was scaled by its *l*_2_ *–*norm. The purpose of this normalization was to optimize the performance of certain classifiers, especially those implemented with the simple Naïve Bayes [61] and Support Vector Machine algorithms [62], [63].

### Design of the experiment

We trained several classifiers to find the best machine learning model for predictively categorizing the corpus documents into the *nano* and *non-nano* classes. We built the following classifiers using the collection of state-of-the-art machine learning algorithms for data mining provided by the Weka workbench (http://www.cs.waikato.ac.nz/ml/weka/) [64]:

1. *Multinomial Naïve Bayes (MNB) classifier*: This variation of the Naïve Bayes classifier, based on a multinomial distribution, is typically used for document classification [65]. Although it makes strong assumptions—e.g. mutually independent variables—it can yield good results in terms of precision and is often used as the simplistic baseline for comparison with other classifier results.
2. *Decision trees (C4.5)*: We built a C4.5 decision tree [66] using the open-source implementation provided by Weka, named J48 [67].
3. *Logistic regression classifiers*: Logistic regression [68] is a method for linear classification that models how the probability of an event can be affected by one or more parameters. We used three different variants of logistic regression:
  - Stochastic Gradient Descent (*SGD*) algorithm for binary class logistic regression [69].
  - Regularized logistic regression, where we applied regularization to the regression parameters to reduce overfitting—a condition which occurs when a classification or estimation model is too complex (too many parameters with respect to the number of observations) since it is trying to over-precisely fit training data instead of learning to generalize.
    - L1-regularized Logistic Regression (*L1-LogReg*) or lasso regression [70], where we used the L1-norm as regularization parameter.
    - L2-regularized Logistic Regression (*L2-LogReg*) or ridge regression [71], where the penalty parameter was the L2-norm.
4. *Support Vector Machines (SVM)*: SVM algorithms [72] have been widely used to model large feature spaces and, particularly, have proven successful when applied to document classification problems [62]. To find the optimal method for our dataset, we compared the performance of several SVM kernel types, obtaining the best performance for the following kernels:
  - *SVM-Lin*: A SVM with a linear kernel.
  - *SVM-Pol*: A SVM with a polynomial kernel of degree 2, using the Sequential Minimal Optimization (SMO) implementation [73], [74].

Independent experiments were run for each classifier. We evaluated their generalization performance on the selected datasets corresponding to the various document representations previously explained: occurrence matrices, frequency matrices, IDF, TFIDF, and their normalized versions.

The entire dataset of 1000 CTs was divided into training and testing sets for which classifiers were tested by 10-fold Cross-Validation (10-fold CV) and Leave-One-Out (LOO) methods. In the case of 10-fold CV, the entire CT summary data set was randomly stratified into ten folds of the same size and each fold was composed of 100 CTs with the same number of *nano* and *non-nano* CTs. For the leave-one-out or LOO tests, we used one CT record from the original body of data as a test example, while the remaining records (999) were used as the training set to develop the model. This was iterated 1000 times by leaving out each of the data items. This type of validation yields a more conservative approach to test the classifier performance [75], [76]. Finally, we also carried out self-consistency tests where the complete body of documents was included in the training set to see if the generated models differed much from the cross-validated ones. These tests give a measure of the stability of the classifier design as a function of the statistical variance across the different training subsets in the cross-validations, in comparison to the over-optimistic performance expected from the over-fitted testing-on-the-training set classifier based on the entire body of 1000 documents.

The validation of the different models was conducted considering several performance measures:

- True Positive *vs*. False Positive rates.
- Precision, for measuring the positive predictive value.
- Recall, which measures the sensitivity.
- F-Measure, which combines precision and recall into a single measure.
- Mathews Correlation Coefficient [77], which measures the correlation between the observed and the predicted class for binary classifiers.
- The AUC, or Area Under the Curve, of the ROC [78], [79], which provides a combined measure of sensitivity and specificity, useful for overall classifier comparison as it integrates results over all possible tradeoffs of decision threshold along the ROC curve.

**Figure 2:**
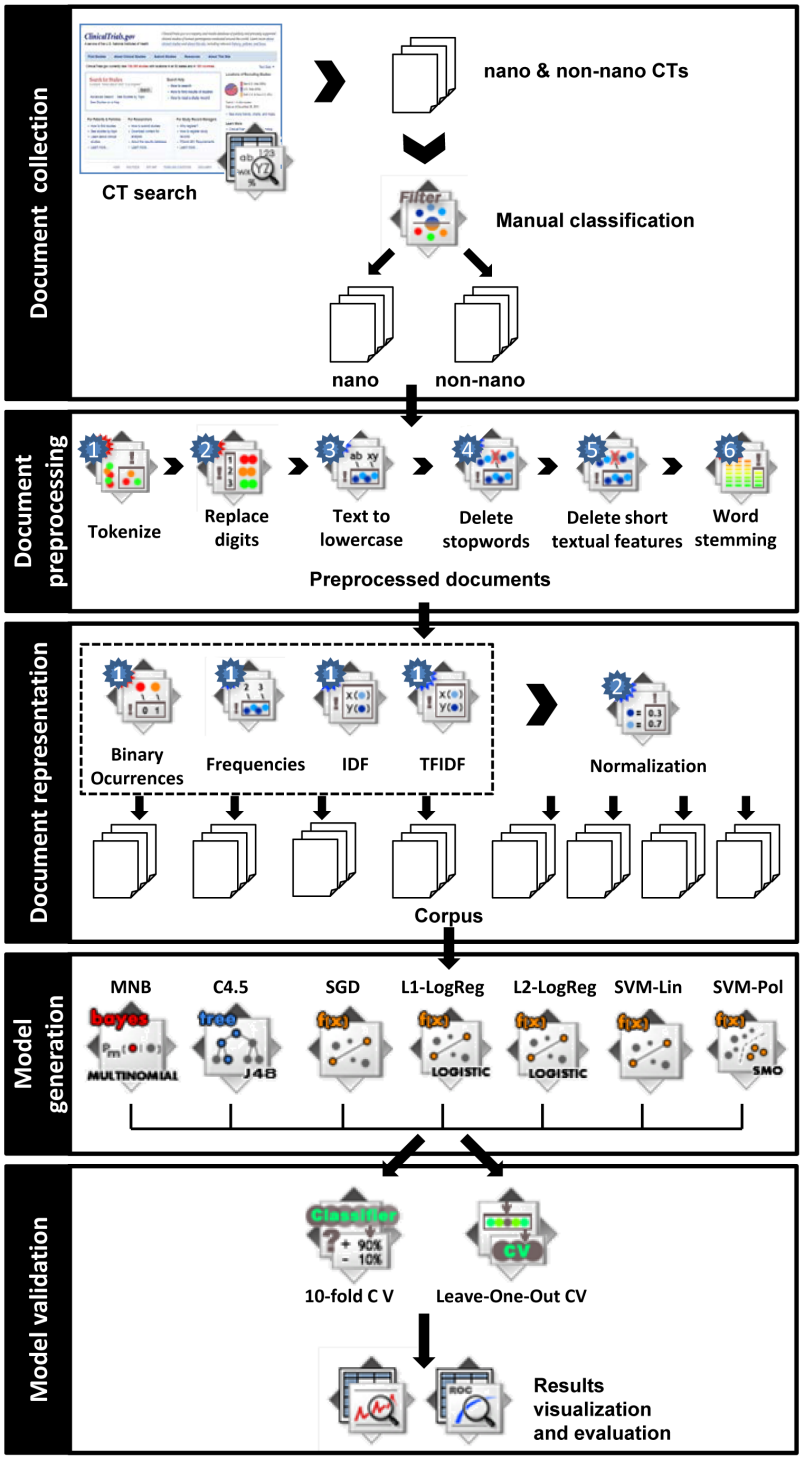
Illustration of the followed approach.

These outcome measures allowed us to evaluate, compare and rank the diverse models generated during our experiments in order to select the best method to address the problem of classifying CTs and validate our approach. Figure 2 presents an overview of the different steps of the method described in this section.

## Results and Evaluation

In this section, we provide a summary of the best results obtained during the above described experiments. Complete results from experiments on unigrams and bigrams are provided in the Supporting Information section (Figures S3 to S5). The reader can find there several comparisons of the results provided for the different document input sets and classifiers.

### Performance

Table 2 presents a comparison of the results provided by the best two learning algorithms under the different transformations using 10-fold CV and LOO, for unigrams. The results yielded by bigram-based models are provided in the Supporting Information Section, since they were outperformed by unigram-based models.

**Table 2:** Best evaluation results obtained in 10-fold and Leave-One-Out cross-validation experiments with unigrams for the different transformations of the input set.

	TP Rate		FP Rate		Precision		Recall		F-Measure		MCC		AUC	
	10-fold CV	LOO	10-fold CV	LOO	10-fold CV	LOO	10-fold CV	LOO	10-fold CV	LOO	10-fold CV	LOO	10-fold CV	LOO
**Binary occurrences**														
L1-LogReg	0,909	0,916	0,091	0,084	0,91	0,917	0,909	0,916	0,909	0,916	0,819	0,833	0,909	0,916
SEM	0,0066	-	0,0066	-	0,0063	-	0,0066	-	0,0066	-	0,0128	-	0,0066	-
SVM-Pol	0,867	0,875	0,133	0,125	0,87	0,878	0,867	0,875	0,867	0,875	0,737	0,753	0,867	0,875
SEM	0,0124	-	0,0124	-	0,0127	-	0,0124	-	0,0124	-	0,0250	-	0,0124	-
**Normalized binary ocurrences**														
SVM-Linear	0,881	0,881	0,119	0,119	0,882	0,881	0,881	0,881	0,881	0,881	0,763	0,762	0,881	0,881
SEM	0,0112	-	0,0112	-	0,0113	-	0,0112	-	0,0112	-	0,0225	-	0,0112	-
SVM-Pol	0,886	0,893	0,114	0,107	0,888	0,894	0,886	0,893	0,886	0,893	0,774	0,787	0,886	0,893
SEM	0,0090	-	0,0090	-	0,0091	-	0,0090	-	0,0090	-	0,0179	-	0,0090	-
**Frequencies**														
L1-LogReg	0,916	0,932	0,084	0,068	0,918	0,934	0,916	0,932	0,916	0,932	0,834	0,866	0,916	0,932
SEM	0,0086	-	0,0086	-	0,0083	-	0,0086	-	0,0086	-	0,0169	-	0,0086	-
L2-LogReg	0,888	0,902	0,112	0,098	0,888	0,903	0,888	0,902	0,888	0,902	0,776	0,805	0,888	0,902
SEM	0,0095	-	0,0095	-	0,0094	-	0,0095	-	0,0096	-	0,0189	-	0,0095	-
**Normalized frequencies**														
SVM-Linear	0,882	0,886	0,118	0,114	0,882	0,886	0,882	0,886	0,882	0,886	0,764	0,772	0,882	0,886
SEM	0,0084	-	0,0084	-	0,0086	-	0,0084	-	0,0084	-	0,0170	-	0,0084	-
SVM-Pol	0,888	0,882	0,112	0,118	0,889	0,884	0,888	0,882	0,888	0,882	0,777	0,766	0,888	0,882
SEM	0,0087	-	0,0087	-	0,0085	-	0,0087	-	0,0088	-	0,0172	-	0,0087	-
**IDF**														
L1-LogReg	0,944	0,955	0,056	0,045	0,946	0,956	0,944	0,955	0,944	0,955	0,89	0,911	0,944	0,955
SEM	0,0086	-	0,0086	-	0,0075	-	0,0086	-	0,0087	-	0,0161	-	0,0086	-
SVM-Linear	0,905	0,908	0,095	0,092	0,906	0,909	0,905	0,908	0,905	0,908	0,811	0,817	0,905	0,908
SEM	0,0109	-	0,0109	-	0,0110	-	0,0109	-	0,0109	-	0,0218	-	0,0109	-
**Normalized IDF**														
L1-LogReg	0,851	0,854	0,149	0,146	0,866	0,866	0,851	0,854	0,849	0,853	0,717	0,72	0,851	0,854
SEM	0,0102	-	0,0102	-	0,0082	-	0,0102	-	0,0107	-	0,0182	-	0,0102	-
SVM-Linear	0,896	0,899	0,104	0,101	0,897	0,899	0,896	0,899	0,896	0,899	0,793	0,798	0,896	0,899
SEM	0,0086	-	0,0086	-	0,0086	-	0,0086	-	0,0086	-	0,0171	-	0,0086	-
**TFIDF**														
L1-LogReg	0,913	0,912	0,087	0,088	0,917	0,917	0,913	0,912	0,913	0,912	0,83	0,829	0,913	0,912
SEM	0,0058	-	0,0058	-	0,0053	-	0,0058	-	0,0058	-	0,0110	-	0,0058	-
L2-LogReg	0,905	0,902	0,095	0,098	0,905	0,902	0,905	0,902	0,905	0,902	0,81	0,804	0,905	0,902
SEM	0,0086	-	0,0086	-	0,0085	-	0,0086	-	0,0086	-	0,0171	-	0,0086	-
**Normalized TFIDF**														
SVM-Linear	0,907	0,91	0,093	0,09	0,909	0,912	0,907	0,91	0,907	0,91	0,816	0,822	0,907	0,91
SEM	0,0068	-	0,0068	-	0,0072	-	0,0068	-	0,0068	-	0,0140	-	0,0068	-
SVM-Pol	0,869	0,87	0,131	0,13	0,885	0,884	0,869	0,87	0,868	0,869	0,754	0,753	0,869	0,87
SEM	0,0084	-	0,0084	-	0,0078	-	0,0084	-	0,0087	-	0,0160	-	0,0084	-

As indication of the variance of the obtained estimations, for 10-fold cross-validation, the table provides the value of the standard error of the mean (SEM) in addition to the figures resulting from the average of the 10 models.

**Figure 3:**
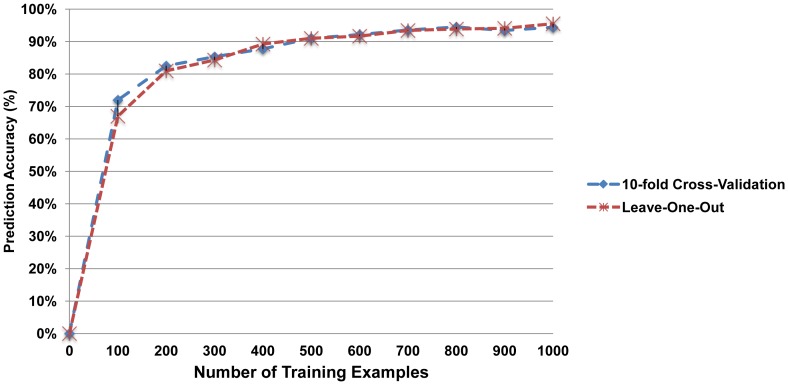
Learning curve for L1-regularized logistic regression in the case of unigrams with IDF transformation.

As shown in the table, in the case of unigrams, the best performance results were obtained using L1-normalized logistic regression with the IDF transformation. This regression model produced the best classifier with 10-fold CV as well as with LOO experiments. Figure 3 shows the learning curve for this best empirical classification model for both types of validation. The learning curve represents the prediction accuracy (percentage of correct results) *vs*. the training set size (number of training examples) and it is useful to see whether the machine learning classifier is suffering from bias or overfitting. The learning curve shows changes in the learning rate as more documents were added to our corpus. For its calculation, we ran several experiments increasing the size of the training set incrementally —creating a 10% training set and 90% test set from the original dataset, then successively reducing the test set until it comprised only 5% of the overall dataset—, and plotted the accuracy as a function of the cardinality of the training set. For the gradual incrementation of the training set size, in the Advanced Mode of the Weka Experimenter, we used the *CrossValidationResultProducer* (for varying the number of instances a classifier is trained on) in conjunction with the *LearningRateResultProducer* (to generate the learning curve results). The learning curve in Figure 3 tends to increase as we add more training examples. This does indeed suggest that the selected algorithm is learning with experience, showing that it is adequately reflecting the pattern of the data set without undue over- or under-fitting.

**Figure 4:**
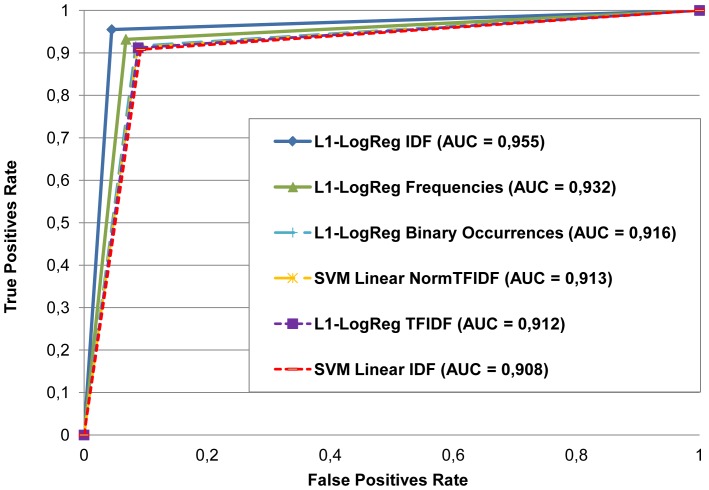
ROC curve for the best classification models resulting from the LOO validation (ranking based on the AUC obtained for each classifier).

On the other hand, Figure 4 shows the ROC curve for a subset of the tested classifiers, including those classification models with the best values for the Area Under the Curve (AUC) measure. The AUC, as an overall threshold-independent statistic to compare classifier performance, indicates the ability of the classifier to distinguish between the *nano* and *non-nano* classes. In addition, Cohen's Kappa statistics [80] for these binary models are given in Table 3. This coefficient measures the reliability of the results reflecting the number of correct results obtained by the classifier not by chance. In the case of the L1-LogReg classifier with IDF transformation, we obtained a value of 0.91 for the kappa statistic, which is close to 1, thus suggesting a high confidence in the reliability of the classifier.

**Table 3:** Kappa statistic for the best classification models resulting from the Leave-One-Out validation.

Classifier	Kappa coefficient
L1-Logistic Regression IDF	0.91
L1-Logistic Regression Frequencies	0.864
L1-Logistic Regression Binary Occurrences	0.832
SVM Linear Normalized TFIDF	0.826
L1-Logistic Regression TFIDF	0.824
SVM Linear IDF	0.816

A comprehensive comparison between the different classification algorithms shows that while SVM-based methods offer good precision results, CT classification is outperformed in most cases by an approach based on logistic regression classifiers, specifically by those using the L1-norm penalty. At the same time, logistic regression has the substantial advantage of producing sparse models, which can be efficiently implemented.

In the case of regularized logistic regression, L1-regularized logistic regression yields consistently higher performance than L2-regularized logistic regression. As reported elsewhere [81], while numerical stability is provided by L2-regularization, L1-regularization yields sparse models and tends to provide better performance for input datasets that include non-discriminating features. In fact, by applying L1-regularized logistic regression we carry out a feature selection process, which considerably improves the results. Finally, when catalyzed by a stochastic gradient descent algorithm, logistic regression eventually outperforms other state-of-the-art algorithms, such as SVM.

For SVM-based models, the linear kernel implementation yielded the best results in most cases. However, we found the SVM polynomial classifier to perform better with normalized datasets. This can be explained due to the fact that SMO implementation we used for the polynomial kernel SVM notably augments its performance with small numeric attributes [73] —i.e. when data is normalized. Conversely, the SVM polynomial kernel offers low performance results when applying the IDF transformation and, more concretely, the TFIDF transformation to non-normalized datasets. This indicates that, in this case, we may be using a too complex algorithm for training our model: the polynomial kernel model contains more information about the training data, but it causes overfitting and does not generalize well to new data. This problem is avoided by normalizing the input data sets, using the normalized versions of IDF and TFIDF, as shown in Figures S3 to S5 in the Supporting Information. Hence, we have smaller values for the features, which correspond to a simpler (smoother) hypothesis that may be easier to generalize to unseen data. Although this regularization prevents overfitting, we can conclude that a simpler model, such as the linear kernel, is more suitable in this case, due to its lower computational cost.

As mentioned previously, we also tested several SVM kernels that are, in general, more flexible than linear and polynomial kernels. For instance, we tested the Radial Basis Function (RBF) kernel, which tends to yield smoother solutions than linear and polynomial kernels, making it more suitable for other type of data rather than text, such as those arising in the classification of images. In fact, the good performance of polynomial kernels relies on the hypothesis that high-order word correlations provide more information than single words. We could not conclude whether this correlation between words prevails or not in our body of documents, but, based on the results, we found that single tokens perform better than bigrams. The use of unigrams outperforms the results obtained with bigrams, which could be due to a lack of correlation between adjacent words. This is consistent with the nature of the textual contents of the CTs classified as *nano*. It should be stressed that, while *nano* CTs contain terms from the nanotechnology field, in most cases, they are acting as simple “modifiers” to biological or medical referents. Therefore, nanomedical terms usually occur as single (though composite) words rather than as bigrams, and tend to be sparse in the text. However, these performance results might also suggest that bigrams require a larger set of documents to obtain significant results than unigrams, since the number of indexing terms for bigrams may be significantly larger —thus introducing noise—, and bigrams are less likely to occur in a document than unigrams —considerably lowering performance for small datasets.

With regards to the other classifiers evaluated in the experiments, as reported elsewhere [65], the multinomial Bayesian (MNB) performance is worse than the state-of-the-art, and thus we just used it as a baseline method for comparison purposes. However, as shown in Figures S3–S5, MNB performs better when using TFIDF scores instead of raw term frequencies [61], especially when no normalization is applied to the length of the resulting feature vectors.

On the other hand, C4.5 decision trees are not usually applied to datasets with a large number of features for efficiency reasons, but they are useful for interpreting the decisions of a classifier involving a conjunction of features. Moreover, in some cases, they yield a good performance—e.g. when the input set has a high number of highly discriminating subsets of subspaces of features. As can be seen in the Supporting Information section, for unigram-based experiments, C4.5 outperforms other classifiers—such as logistic regression or polynomial SVM— for different transformations of the documents vector. Conversely, for bigrams, it yields the worst performance results for the most of the experiments. This is consistent with the earlier comment on the nature of the “nano” term being a common modifier of biomedical referents in the text, making the resulting composite words sufficiently powerful discriminating unigrams for the analysis, so that adjacent words simply add confusing "noise" to the signal that then must be detected during the classifier's learning phase.

It is also significant that feature transformation dramatically improves the performance of the different methods in several experiments. Regarding the different representations of the input dataset, IDF provides the best results, suggesting that while the local weight of a term is a good discriminant, its global weight usually provides a better discriminating performance. With respect to normalization, the SVM linear classifier yields better results for normalized data, while L1- and L2-normalized logistic regression offers better performance without normalization. While normalization tends to improve performance results for most experiments, this is not the case for regularized logistic regression, where normalization appears to inject noise into the dataset.

### Computational cost

In terms of the computational cost of the different classification models, we can make a few observations. Despite the fact that SVM algorithms achieved the state-of-the-art performance, they have a high computational cost, especially when using SVM non-linear kernel classifiers, which are much more complex computationally than linear kernels. In our study, the SVM linear classifier shows close-enough performance to the polynomial kernel classifier regardless of the transformations applied to the input dataset with a much lower computational cost.

However, logistic regression yields better results in terms of computational efficiency. Compared to SVMs, the drawback of logistic regression is that it usually requires an expensive exponential function evaluation during the numerical optimization. But, as suggested elsewhere [82], [83], L1-regularized logistic regression can outperform more recent algorithms for a wide range of classification tasks—as it did for our classification problem—and, in addition, it produces sparse models—with many zero regression parameters— thus reducing the computational complexity. On the contrary, L2-norm provides dense solutions, increasing the computational cost of the classification.

In summary, in this series of experiments, L1-regularized logistic regression and SVMs produce similar results and significantly outperform the rest of the tested classifiers. We can conclude that, as far as computational cost and performance trade-off are concerned, the L1-norm logistic regression emerges as the best choice for our CT summary document classification problem.

## Discussion

This paper makes an original contribution to the design, modeling and analysis of the nanomedicine domain in terms of showing that one can automatically detect the relevance to a nano-related target from a CT summary in ClinicalTrials.gov. We have created an annotated body of nanomedicine CTs, with training and testing sets that can be used to develop extended computational applications for supporting research in the nanomedicine field (see Table S1 within the Supporting Information). To the best of our knowledge, there is no such publicly available reference dataset for clinical nanomedicine. Our approach has produced promising results: given a subset of CTs extracted from ClinicalTrials.gov, our method can be reliably used for automatically determining whether the CT involves the use of nanodrugs. We identified an algorithm (L1-regularized logistic regression) able to deal with such a high-dimensional problem, both in terms of classification performance and computational cost. Although the classification results we obtained in this study are not directly comparable to those resulting by other similar state-of-the-art studies—since the latter are focused on different domains and resort to different training and test sets, in general, our results (F  =  0.955) outperform the results from other recent experiments—that range in the interval [0.85, 0.96] by F-measure—as reported elsewhere [65], [84]–[89]. To our knowledge, these results are the first application of text mining to extract information about nanodrugs and nanodevices from ClinicalTrials.gov, excluding the NanoSifter [90], which covers the dendrimer domain alone.

There are a number of reasons that justify performing such a categorization of CTs into the *nano* vs. *non-nano categories*. These include, for instance, comparing legacy formulations with nanotechnology-based formulations —in terms of aspects such as structure, function, toxicology, pharmacokinetics and pharmacodynamics (PK/PD), clinical immunogenicity, safety and effectiveness—, which would provide additional information to researchers in the nano domain. This knowledge could lead to the reuse of existing products that could be manufactured at the nanoscale and, therefore, re-classified as nanotechnology once this is done. In most cases, current CTs on nanodrugs have not revealed unknown side effects due to the nanoparticle or any of its constituents. Yet, earlier abandoned therapeutics agents that have now been reformulated as nanodrugs are presenting toxicity and side effects due to the special physicochemical properties acquired during the nanomanufacturing process that were not considered during the design of the original drug [6], [91]. While safety and efficacy trials will, of course, still remain essential, our approach could considerably simplify and reduce the steps involved with the need to pursue, as currently, assays and clinical trials, by instead extrapolating clinical data and using modeling and simulation tools from the related prior experiments.

In addition, physicians —and concerned patients— are currently increasingly interested in CTs on *non-nano* drugs, since they often seek information about diagnosis and/or therapy for a given disease. On the other hand, most *nano* CTs currently archived in ClinicalTrials.gov are in an early stage —either phase I or phase II—, being those more targeted at clinicians and researchers —and even pharmaceutical and nanotechnology companies— who are more interested in research or the mid-term or long-term applications of nanodrugs. In addition, different users will likely search for different information. For instance, *nano* users often search for information about the composition and characterization (e.g. size, cytotoxicity, ligands, hydrosolubility, bioavailability, pharmacokinetics etc.) of the nanocompound, while physicians are more interested in the patient profile (e.g. sex, race, age, etc.), drug dosage, study arms, etc. Knowing in advance the category to which a given CT belongs, would ease the way information is indexed, searched and presented to users based on their likely interests and goals, which could be deduced or inferred from their profiles.

The information about *nano* CTs available in ClinicalTrials.gov —although the same applies to other existing registries such as those cited in the introduction— is not currently connected to other repositories involving related data, such as physicochemical properties (caNanoLab, https://cananolab.nci.nih.gov/caNanoLab/), biological interactions (Nanomaterial-Biological Interactions Knowledgebase, http://nbi.oregonstate.edu/), normalized vocabularies and ontologies (the Unified Medical Language System, http://www.nlm.nih.gov/research/umls/, and the NanoParticle Ontology, http://www.nano-ontology.org/), environmental and health safety data (the Nanomaterial Registry, https://www.nanomaterialregistry.org/), modeling and simulation experiments (nanoHub, http://nanohub.org/), etc. We believe that once the *nano* CTs have been automatically identified, it is possible to establish links among related information —either manually or automatically, using artificial intelligence techniques—, in the same manner as with most NCBI repositories (http://www.ncbi.nlm.nih.gov/). Furthermore, the integrated information could be exploited to compare and curate experimental results that are currently distributed in different databases. We believe that this rationale supports the initiative of automatically labeling the existing CTs in the ClinicalTrials.gov database so as to support, assist, and encourage future research in nanomedicine.

Regarding the limitations of the approach presented here, we have consulted different CT registries as well as drug registries, Pharma websites and other information sources and, unfortunately, we have run into several barriers, especially related to the unavailability of public clinical nanomaterial data. We have also identified several issues concerning the format and nature of the data reported in CTs. First, the current identification system of CTs has not been agreed to by consensus, and the main registries are not fully committed to use a common coding system. Second, drug nomenclature does not follow a standard, and a large number of synonyms can be found to designate the same compound, especially in the nanomedicine area. Finally, CT summaries do not report the same type of information and do not have a common structure in terms of text analysis. All of these issues add complexity to the task of automatically parsing clinical summaries and result reports —and it is worth noting that a similar situation arises when analyzing the scientific literature. This fact could constrain the application of the statistical approach presented in this paper, since it relies on common patterns and terms that were found in the documents.

Nevertheless, we believe that our work can stimulate a wide range of novel computational applications to support nanomedical research. An interesting example of application is the automated creation of a repository linking nanoparticles and/or nanodevices to side-effects reported in the CTs, an idea that we have already explored [92]. Once an automated CT tagger—i.e. a classification model that tags CTs as *nano* or *non-nano*, like the one presented in this paper—is available, it is possible to reliably apply text mining techniques to extract relevant information. This includes but is not limited to— nanoparticles and nanodevices names and formulations, their potential side-effects, routes of exposure, etc. We are currently conducting an analysis of the distribution of the nanomedical concepts patterns found in the dataset and their relationships, as well as working on the development of a CT information retrieval system based on the results obtained from this work. In addition to CT summaries, this approach could be applied to the vast nanomedical literature and also adapted to extract data from other textual sources.

## Conclusions

With the volume of experimental and clinical data related to nanomedicine increasing rapidly, manual analysis and annotation of studies on nanodrugs has become slow and largely impractical. In this context, the development of automatic approaches targeted at discriminating information from the *nano* and *non-nano* domains becomes necessary. In this paper, we have presented two original contributions to the nanoinformatics field. First, we have created a training and testing set for a binary textual classification problem targeted at identifying previously unseen CTs as being *nano* or *non-nano*. Second, we have conducted a thorough review of the state of the art both on machine learning-based techniques for binary document categorization and existing repositories of drugs and registries of CTs. We selected the classification methods and algorithms reported in the literature as the best performers for binary text categorization problems and applied these methods to the training and test sets we created. We selected the most efficient method to classify CTs into the *nano* and *non-nano* categories, thus producing categorization models whose results outperform most state-of-the-art classifiers. We believe that such a classifier can help catalyze the research in translational nanomedicine, thus enabling a wide range of applications that cannot be addressed well with a raw repository of unclassified CTs.

The analysis of clinical trials related to nanomedicine, carried out by integrating reported results over all the different available databases worldwide, could result in the extraction of potential correlations, and new patterns and trends in nanomedical data. The analysis of correlations between multiple pre-clinical and clinical studies may be of value in areas such as nanotoxicity and targeted drug therapy, where certain underlying patterns and trends could support inferences that inform future research in nanomedicine. By way of an example, results could serve to compare new formulations with existing ones and determine additional side effects that may arise due to the newly added components and/or the manufacturing process (i.e. to the application of nanotechnology to the original drug). This work could also facilitate researchers in automatically discovering new knowledge from CTs such as, for instance, uncovering potential toxicity of novel nanodrugs or recruiting patients who are most likely to respond positively to a certain nanoparticle intervention due to their participation in earlier CTs using similar drugs.

Shorter time-to-market cycles for nanodrugs and medical nanodevices require researchers to act on insights faster than ever, as well as for computer scientists to develop new methods and tools to efficiently manage this new knowledge, providing users with the necessary processing and analysis capacity. Furthermore, publishers, governmental agencies and the Pharma industry will surely need to develop new open data strategies and the setting of standards for CT data. This study points out that valuable data on nanomedical CTs are already available implicitly within the ClincalTrials.gov repository, and that machine learning methods can be used to combine the values of individual word-features from the CT summaries into a predictor for detecting *nano*-related CTs. These kinds of approaches are needed to help gather, organize and integrate the huge volume of existing data which is potentially relevant for nanomedicine—including pre-clinical and clinical data—and make them accessible to researchers [93].

## Acknowledgments

The authors would like to thank Prof. Alejandro Pazos and Prof. Julián Dorado, from the University of A Coruña (Spain), who provided invaluable insight and expertise for assessing and curating the extensive body of material used in this research.

## Supporting Information

Figure S1 **Boxplot of document length for unigrams.** The red band inside the box represents the median of the distribution of unigrams per document (522,875 unigrams).
(TIF)

Figure S2 **Boxplot of document length for bigrams.** The red band inside the box represents the median of the distribution of bigrams per document (297,667 bigrams).
(TIF)

Figure S3 **Precision results for the input set under different transformations and classifiers, with 10-fold Cross-Validation and Leave-One-Out Cross-Validation, for both unigrams and bigrams.**
(TIF)

Figure S4 **F-Measure for the input set under different transformations and classifiers, with 10-fold Cross-Validation and Leave-One-Out Cross-Validation, for both unigrams and bigrams.**
(TIF)

Figure S5 **MCC for the input set under different transformations and classifiers, with 10-fold Cross-Validation and Leave-One-Out Cross-Validation, both for unigrams and bigrams.**
(TIF)

Table S1 **Results obtained from the manual classification of the summaries extracted from ClinicalTrials.gov.** Each document belonging to the set—identified by its National Clinical Trial (NCT) number—was manually tagged as being either “nano-related” (500 *nano* CTs) or “non nano-related” (500 *non-nano* CTs).
(XLSX)
