# Supplementary figures and images for: A Machine Learning Approach to Identify Clinical Trials Involving Nanodrugs and Nanodevices from ClinicalTrials.gov
Source: PLoS One. 2014 Oct 27;9(10):e110331. doi: 10.1371/journal.pone.0110331 (PMC4210133; doi:10.1371/journal.pone.0110331)

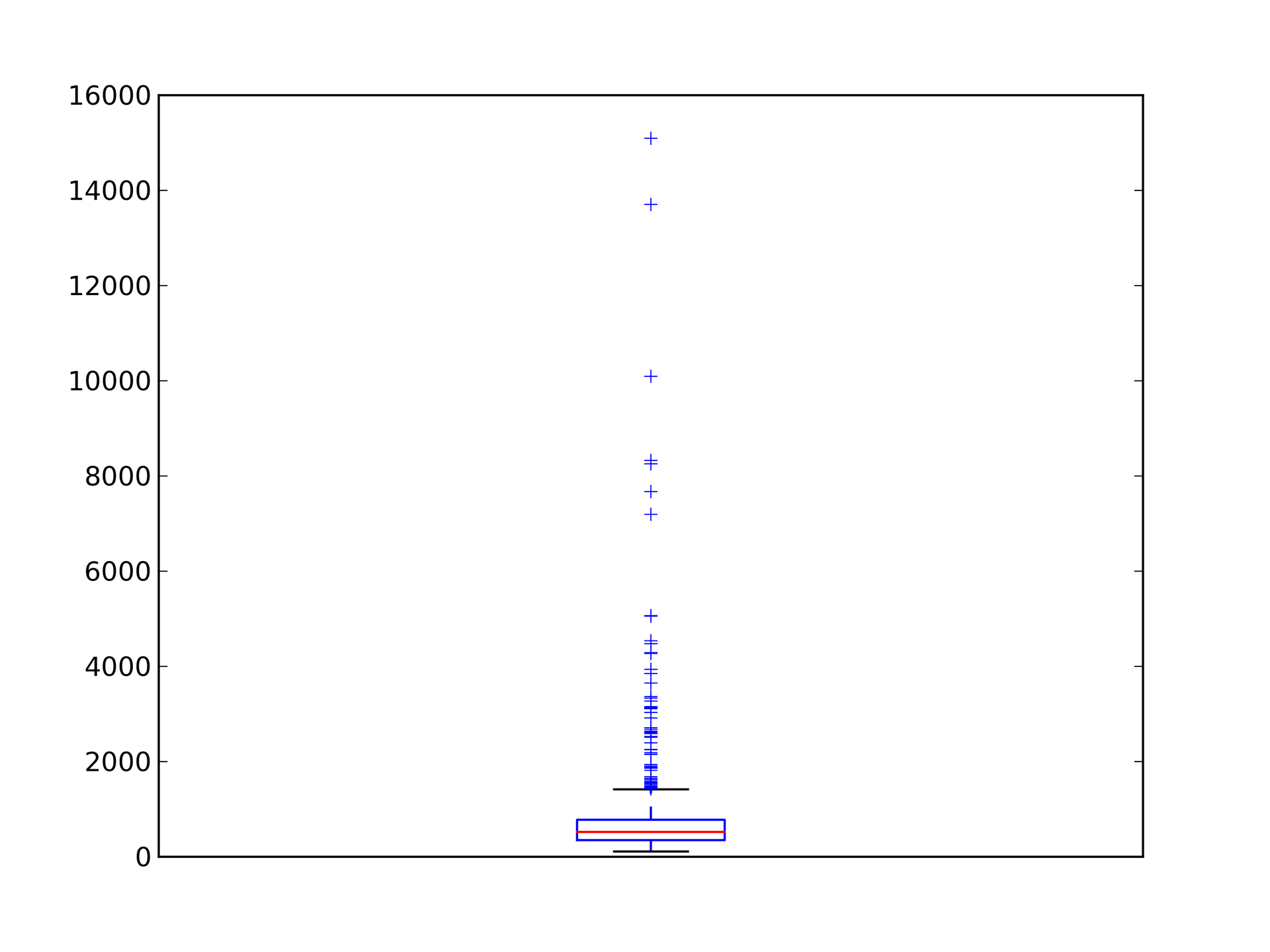

Supplement: Figure S1 — Boxplot of document length for unigrams. The red band inside the box represents the median of the distribution of unigrams per document (522,875 unigrams). (TIF) [file pone.0110331.s001.tif]

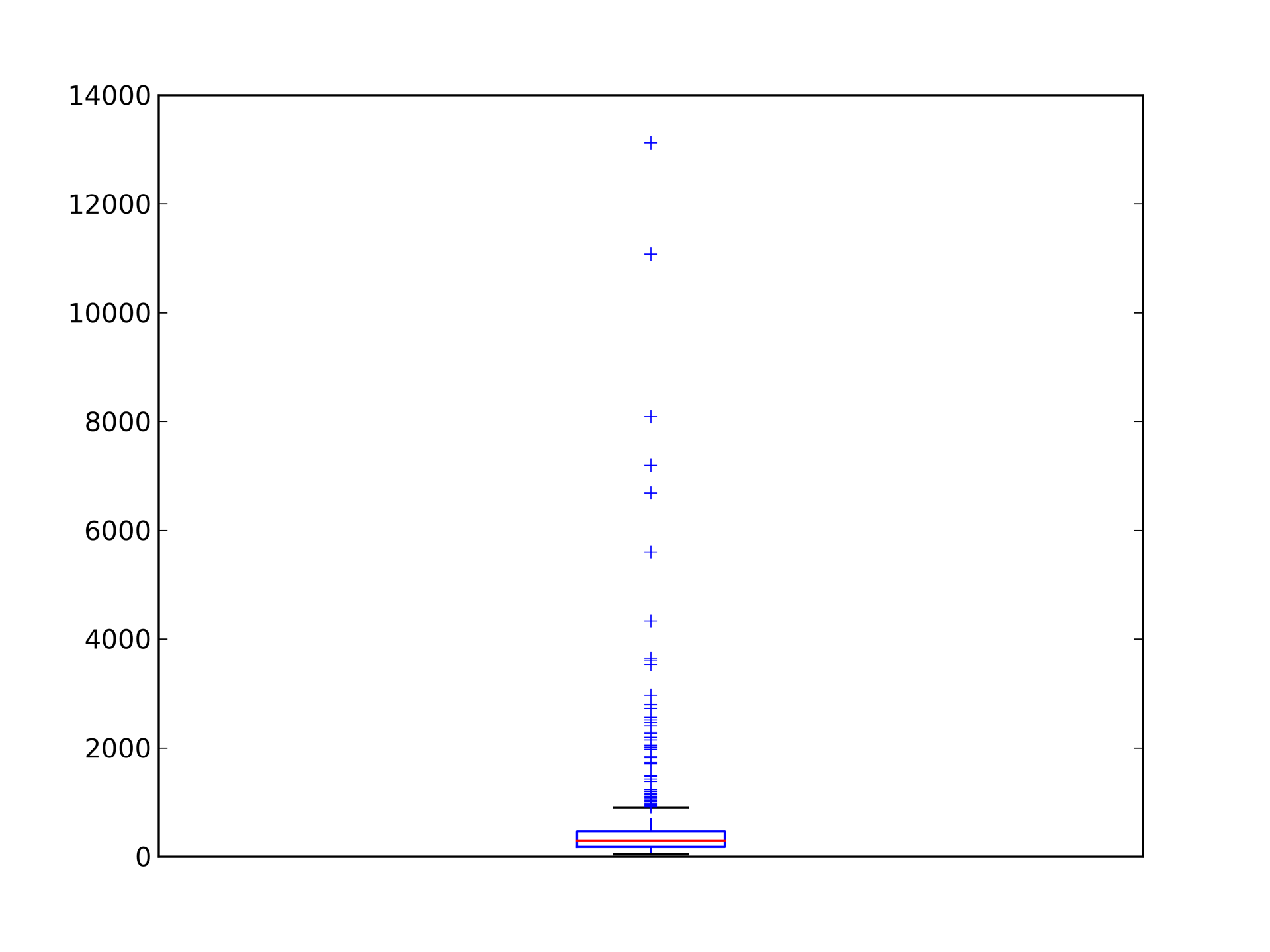

Supplement: Figure S2 — Boxplot of document length for bigrams. The red band inside the box represents the median of the distribution of bigrams per document (297,667 bigrams). (TIF) [file pone.0110331.s002.tif]

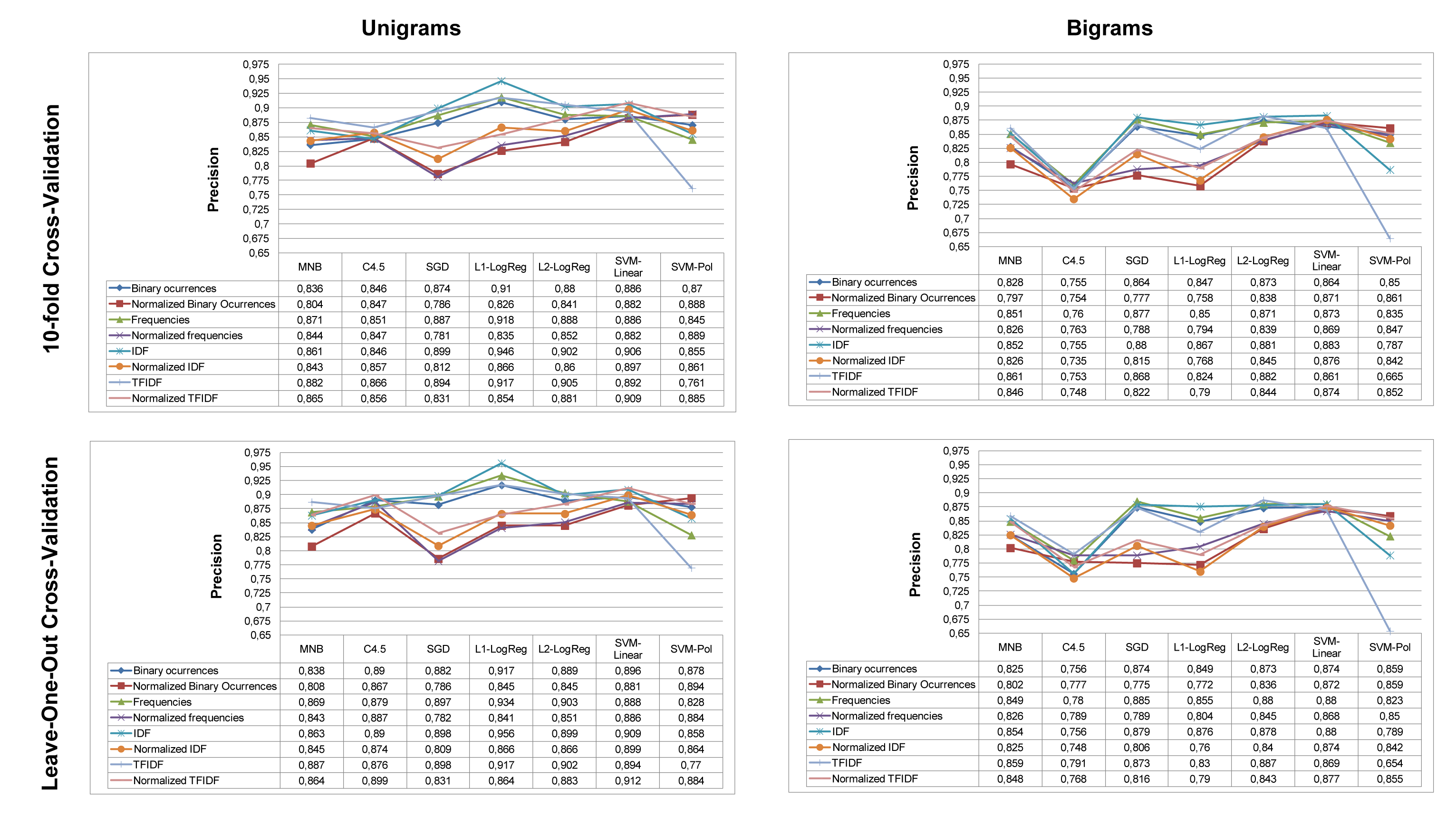

Supplement: Figure S3 — Precision results for the input set under different transformations and classifiers, with 10-fold Cross-Validation and Leave-One-Out Cross-Validation, for both unigrams and bigrams. (TIF) [file pone.0110331.s003.tif]

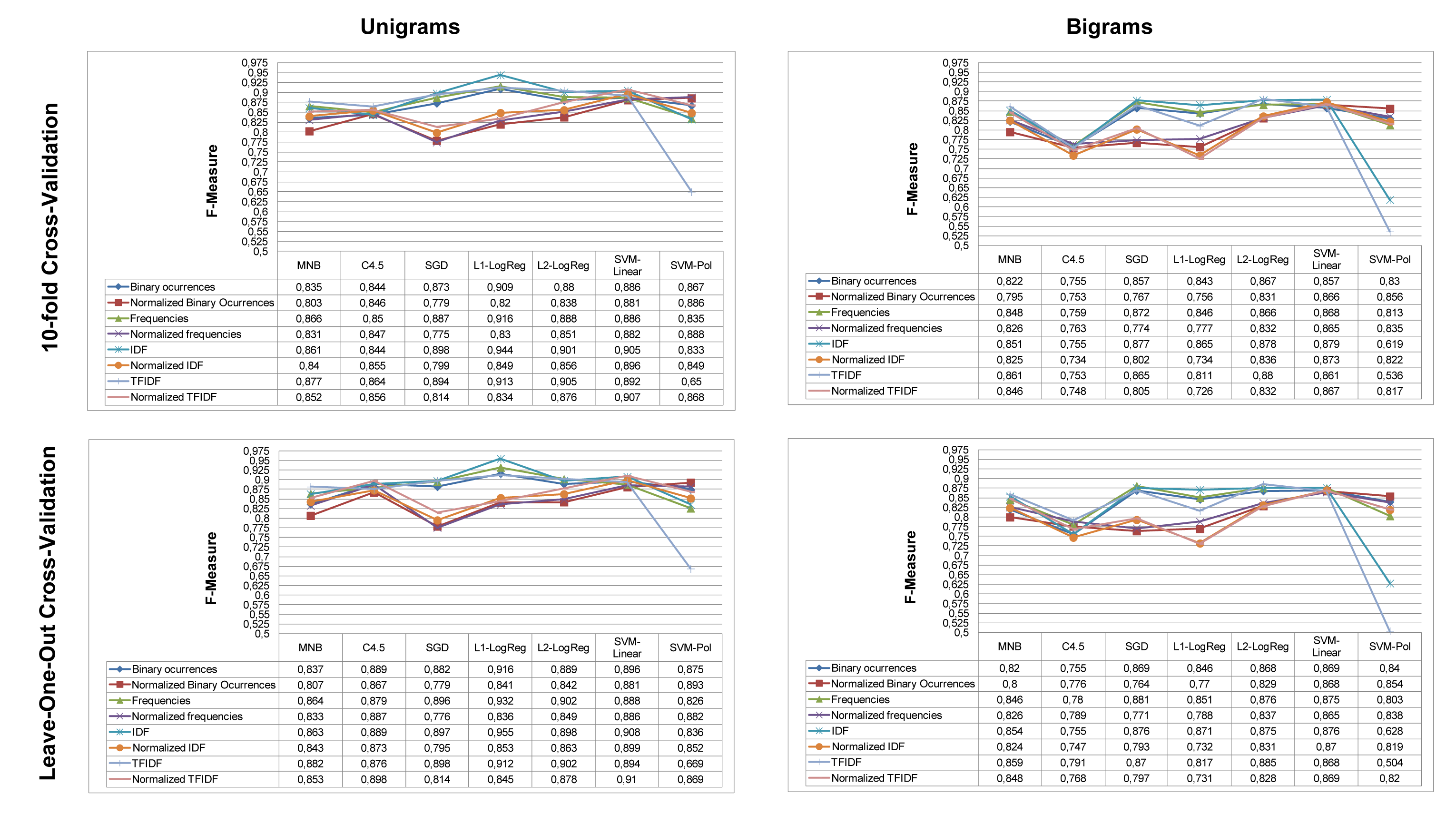

Supplement: Figure S4 — F-Measure for the input set under different transformations and classifiers, with 10-fold Cross-Validation and Leave-One-Out Cross-Validation, for both unigrams and bigrams. (TIF) [file pone.0110331.s004.tif]

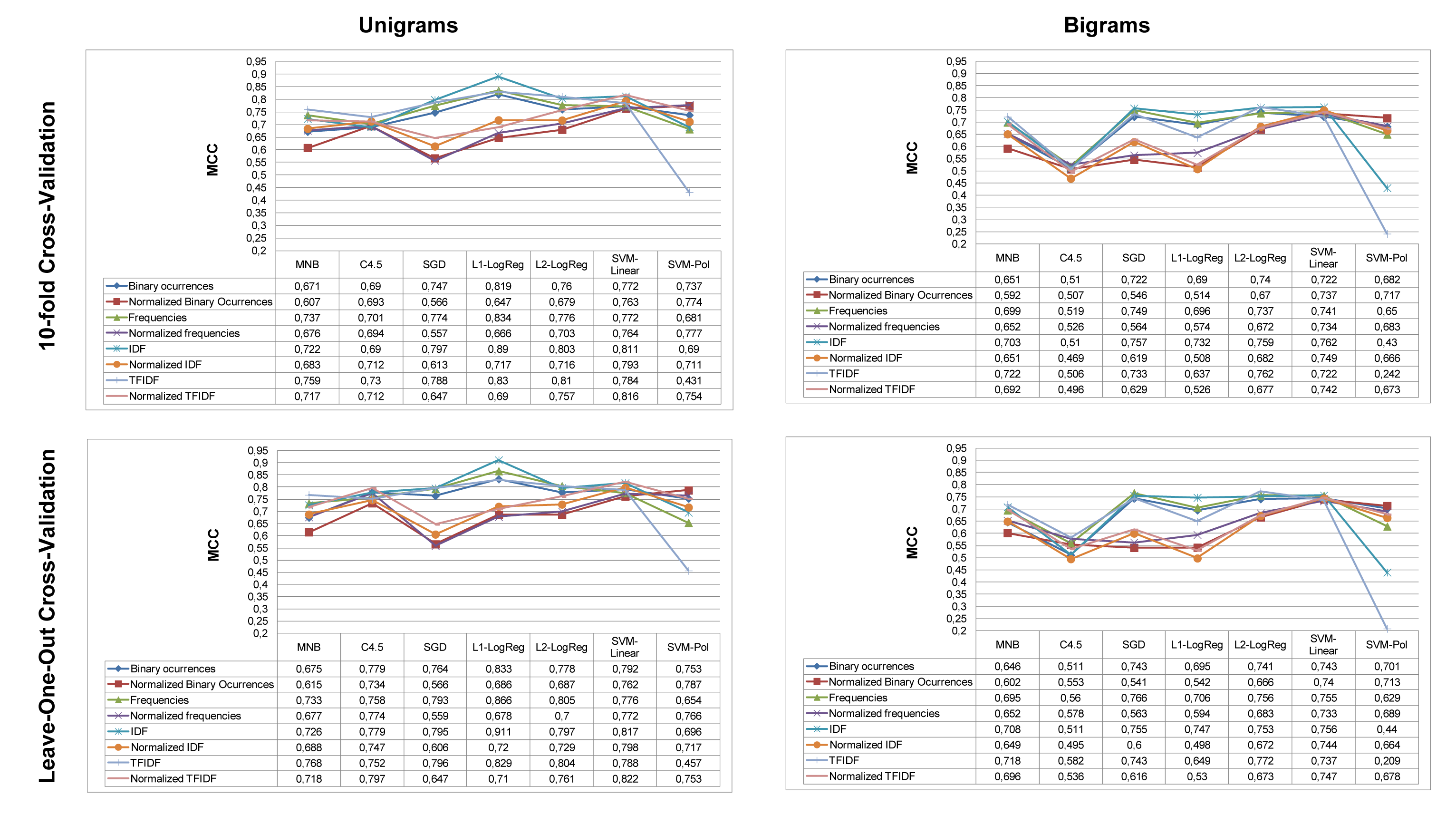

Supplement: Figure S5 — MCC for the input set under different transformations and classifiers, with 10-fold Cross-Validation and Leave-One-Out Cross-Validation, both for unigrams and bigrams. (TIF) [file pone.0110331.s005.tif]
